# DNA Methylation Age Is More Closely Associated With Infection Risk Than Chronological Age in Kidney Transplant Recipients

**DOI:** 10.1097/TXD.0000000000001020

**Published:** 2020-07-15

**Authors:** Joanna Schaenman, Xinkai Zhou, Rong Guo, Maura Rossetti, Emily C. Liang, Erik Lum, Basmah Abdalla, Suphamai Bunnapradist, Phuong-Thu T. Pham, Gabriel Danovitch, Arun Karlamangla, Elaine Reed, Steve Horvath, David Elashoff

**Affiliations:** 1 Department of Medicine, Division of Infectious Diseases, David Geffen School of Medicine at UCLA, Los Angeles, CA.; 2 Division of General Internal Medicine and Health Services Research, David Geffen School of Medicine at UCLA, Los Angeles, CA.; 3 Department of Pathology and Laboratory Medicine, UCLA Immunogenetics Center, David Geffen School of Medicine at UCLA, Los Angeles, CA.; 4 Department of Medicine, Division of Nephrology, David Geffen School of Medicine at UCLA, Los Angeles, CA.; 5 Department of Medicine, Division of Geriatrics, David Geffen School of Medicine at UCLA, Los Angeles, CA.; 6 Department of Human Genetics, David Geffen School of Medicine at UCLA, Los Angeles, CA.

## Abstract

Older kidney transplant recipients demonstrate increased rates of infection but decreased rates of rejection compared with younger recipients, suggesting that older transplant patients are functionally overimmunosuppressed. We hypothesized that this is a consequence of reduction in immunological activity due to biological aging and that an immune biological age, as determined by DNA methylation (DNAm), would be associated more strongly with incidence of infection than chronological age.

**Methods.:**

DNAm analysis was performed on peripheral blood mononuclear cell collected from 60 kidney transplant recipients representing older (≥age 60 y) and younger (aged 30–59 y) patients 3 months after transplantation. DNAm age was calculated based on methylation status of a panel of CpG sites, which have been previously identified as indicative of biological age.

**Results.:**

Correlation was seen between chronological and DNAm age; however, there were many patients with significant differences (either acceleration or slowing) between DNAm age and chronological age. A statistically significant association was seen between increased DNAm age and incidence of infection in the first year after kidney transplantation, whereas no significant association was seen between chronological age and infection.

**Conclusions.:**

Assessment of DNAm age holds promise as an approach for patient evaluation and individualization of immune suppression regimens. This analysis may provide insights into the immunological mechanism behind increased incidence of infection observed in older transplant patients. The ability to measure biological age would allow for patient risk stratification and individualization of immunosuppression, improving outcomes for the growing numbers of older patients undergoing kidney transplantation.

## Abstract 2

Supplemental Digital Content is available in the text.

## INTRODUCTION

The numbers of older patients with chronic kidney disease undergoing kidney transplantation continues to grow. However, older transplant recipients experience increased rates of infection, malignancy, and death, and lower rates of rejection, compared with younger patients.^1-4^ This suggests that immune dysfunction is a potential mechanism behind the vulnerability of the older transplant recipient to adverse outcomes. However, without a method for assessing a patient’s level of immunosenescence, it is difficult to move beyond the current “one-size-fits-all” approach to immunosuppression.^5-7^

Attempts to adjust immunosuppression based on chronological age have not been successful and have led to increased incidence of rejection,^6^ suggesting the need to measure a patient’s immune system “biologic age” for patient risk stratification and individualization of immunosuppression regimens.

Because T-cell immunosenescence is known to be associated with increased vulnerability to infection and malignancy in older adults, we hypothesized that measurement of DNA methylation (DNAm) state of peripheral blood would be the ideal approach for determining the biological age of a patient’s immune system. This hypothesis is supported by our previous work demonstrating that peripheral blood measurement of immune dysfunction is associated with increased patient age and incidence of infection after kidney transplantation and the work of others demonstrating association between increase in immunosenescence and decreased incidence of rejection after kidney transplantation.^1,8,9^ These differences are also reflected in increased expression of proinflammatory transcripts and decreased expression of adaptive immunity-related transcripts in older as compared with younger kidney transplant recipients.^10^

Calculation of biological age via DNAm analysis of peripheral blood is a method well suited to fill this need. DNAm or epigenetic analysis has been used to reliably measure biological age: analysis of DNAm of a set of specific genetic loci has been shown to reliably predict aging in various tissues.^11-13^ Decreases in methylation levels at these loci allow increased access to transcriptional machinery and are correlated with changes in gene expression.^14,15^ These epigenetic changes are known to influence T-cell differentiation.^16^ This measure of biological age, the so-called “epigenetic clock,” can be calculated from a validated subset of comethylation sites and has been found to be a better predictor of age-associated diseases than chronological age, and to be strongly associated with declines in physical health and all-cause mortality.^12,17^

Accurately assessing patient biological and, specifically, immunological age by measuring DNAm levels, therefore, offers the ability to determine patient risk for infection after transplantation and to individualize immunosuppression regimens using a precision measurement approach. This analysis additionally offers insight into the mechanism behind the immune dysfunction and vulnerability to adverse clinical outcomes in the older transplant recipient.

We present here an analysis of DNAm in a cohort of older and younger kidney transplant patients demonstrating the potential benefit of epigenetic age analysis in the context of kidney transplantation.

## MATERIALS AND METHODS

### Clinical Care

We enrolled kidney transplant recipients after transplantation at Ronald Reagan Medical Center. The University of California, Los Angeles (UCLA), Institutional Review Board approved this observational study. All patients signed informed consent. As described previously, inclusion criteria were any adult kidney transplant recipient willing and able to provide informed consent, and exclusion criteria were the presence of active infection or rejection at the time of blood collection.^18^ Blood was collected for peripheral blood mononuclear cell (PBMC) isolation at 3 months after transplantation during an outpatient clinic visit, as previously described. The 3-month time point was chosen as this is the point reliably representing recovery of lymphocyte count, making PBMC collection more feasible. We identified 24 older patients (aged ≥60 y) who had PBMC available for analysis; these were cohort matched with 1–2 younger patients as feasible on donor type (deceased versus living) and induction type (antithymocyte globulin [ATG] versus basiliximab), for a total cohort of 60 patients. Age 60 years rather than 65 years is commonly used as a cutoff for older patients in the context of solid organ transplantation, given the increased frequency of comorbidities compared with the general population. Details of immunosuppression and antibiotic prophylaxis were previously described.^18^ Patients received similar maintenance immunosuppression regimens with protocolized target drug levels and monitoring for infection. Mycophenolate mofetil daily dose, tacrolimus trough, and glomerular filtration rate were assessed at the time of sample collection. Patients were characterized as having infection, rejection, or no events during the first year after transplantation based on chart review using standard clinical criteria as previously reported.^18^ The overall infection group included those with bacteremia, pneumonia, respiratory or systemic viral infections requiring hospitalization, or cytomegalovirus viremia leading to antiviral therapy. Urinary tract infections were not included in this analysis, given the difficulty in defining these infections via chart review. For patients experiencing both infection and rejection during the first year, only the earlier occurring event was counted.

### DNA Methylation Analysis

PBMC were isolated and frozen for storage using standard techniques.^19^ Genomic DNA was isolated by the UCLA Biological Samples Processing Core using the Autopure LS nucleic acid purification instrument (Gentra Systems) to extract DNA, followed by purity analysis and quantitation using optical density 260/280. Genomic DNA was then bisulfate converted using the EZ-methylation kit (Zymo Research) and processed by the Illumina Infinium whole-genome genotyping protocol followed by hybridization to Infinium MethylationEPIC BeadChip arrays in the UCLA Neuroscience Genomics Core Laboratory, a full-service core facility specializing in gene expression and DNAm array analysis. In total, 866 297 CpG sites were analyzed. Methylation levels were quantified using ratio of intensities between methylated and unmethylated alleles using previously published methods.^20^ Standard quality assurance metrics were applied to ensure validity of results.

DNAm data were used for biological age determination using the published calculator for the Infinium MethylationEPIC BeadChip, which is based on a subset of 292 CpG sites identified as predictive of age-associated disease and mortality.^13^ This approach is an algorithmic computation based on the DNAm data, which has been well-characterized in a number of different tissue types including peripheral blood and has demonstrated excellent predictive value for age-associated co-morbid conditions and mortality.^12-14^

### Statistical Analysis

Patient DNAm data were normalized using beta-mixture quantile method before DNAm age was calculated based on the Horvath method.^13^ Two age acceleration measures were obtained, difference between chronological and DNAm age (age acceleration difference) and the residual from a linear model of regressing DNAm age on chronological age (age acceleration residual).

Descriptive statistics were reported to summarize demographic and clinical characteristics, mean (STD) or median (min-max) for numerical variables, and N (%) for categorical variables. These demographic and clinical characteristics were compared between age groups defined by chronological age, DNAm age, and age acceleration (negative versus positive) using 2-sample *t*-test or Wilcoxon test for numerical variables, Pearson, Fisher’s exact test, or Chi-square test for categorical variables, as appropriate. Kaplan-Meier analysis was performed for time-dependent analyses of infection or rejection, with statistical analysis by Gray’s test to evaluate hypotheses of equality of cumulative incidence functions between 2 groups. *P* values <0.05 were considered statistically significant. All statistical analyses were performed using R 3.4.1 or SAS 9.4 (SAS Institute Inc., Cary, NC).

## RESULTS

### Demographic Characteristics and Clinical Outcomes Based on Chronological Age

Our sample consisted of 24 kidney transplant recipients aged 60 years or older and 36 younger patients (Table 1). There were similar distributions of sex, race, and ethnicity, and similar incidence of induction with ATG (29% versus 28%) deceased kidney donor (46% versus 47%) in the chronologically older and the younger patient groups (Table 1).

**TABLE 1.**
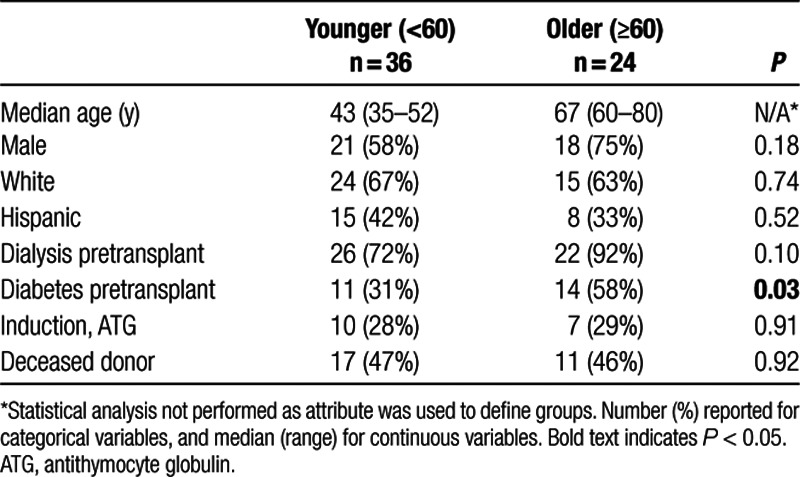
Demographic and clinical characteristics of older and younger kidney transplant recipients matched on transplant type and induction

A total of 25 patients had infections in the first year after transplantation, and 8 experienced allograft rejection. Rates of infection and rejection were not significantly different between the older and younger transplant recipients (Table 2).

**TABLE 2.**
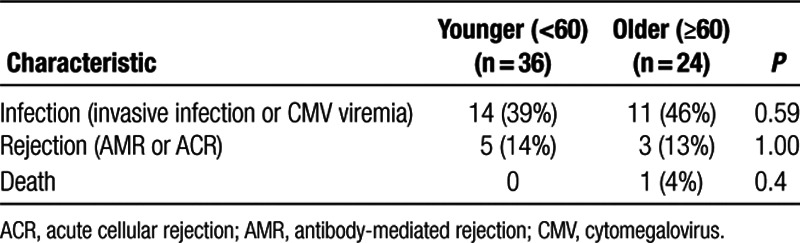
Association between chronological age and clinical outcomes of infection and rejection after kidney transplantation

### Analysis of DNA Methylation Age and Association With Demographic and Health Characteristics

**FIGURE 1.**
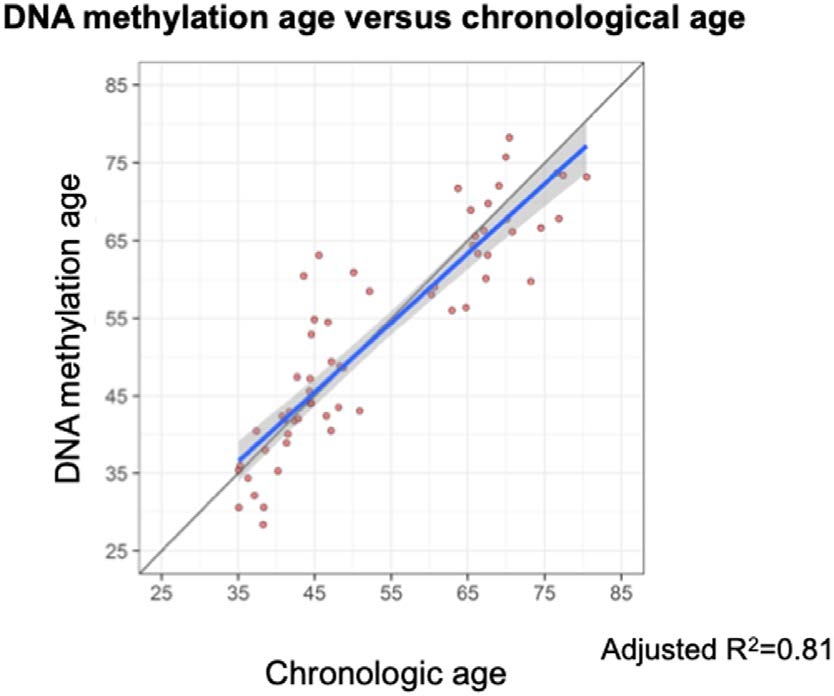
Linear regression of DNAm age on chronological age. DNAm age was calculated from the 292 CpG sites using BMIQ normalization. The gray line represents a diagonal line of Y = X, while the blue represents the regression line. The gray shade indicates the 95% prediction interval. DNAm, DNA methylation; BMIQ, beta-mixture quantile.

DNAm or epigenetic age was calculated for each patient based on methylation patterns at a defined subset of 292 CpG sites.^13^ Linear regression analysis demonstrated a linear correlation between patient chronological age and the calculated DNAm age (*P* < 0.001; adjusted R^2^ = 0.81, Figure 1). Despite the overall correlation observed, review of individual patient data points revealed many discrepancies between chronological age and DNAm age, some patients appeared below the regression line with negative regression residual, indicating retardation of DNAm age compared with chronological age. In contrast, some patients appeared above the regression line with positive residual, demonstrating acceleration of DNAm age compared with chronological age. These discrepancies on the residual were not significantly associated with chronological patient age.

Calculated DNAm age ranged from 28 to 78 years in our cohort of patients with chronological age from 35 to 80 years old. Median DNAm age was 53.6 years. Twenty-four patients in the sample were chronologically 60 years or older, and 22 patients had DNAm age 60 years or greater. Analysis of demographic characteristics based on older compared with younger DNAm age (≥60 compared with <60) did not show any significant difference by demographic characteristics (Table 3). There were similar percentages of sex, race, and ethnicity, and similar percentages of induction with ATG and deceased kidney donor in the older and the younger patient groups as classified by DNAm age (Table 3).

**TABLE 3.**
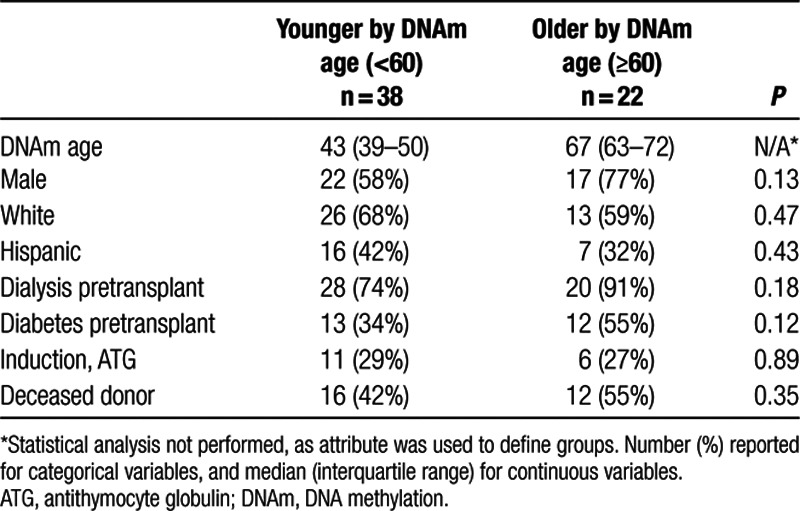
Association between demographic/health characteristics and DNAm age

### Analysis of Clinical Outcomes by DNAm Age

Median DNAm age was 56 years in patients experiencing infection compared with 54 years in those without infection in the first year. Analysis comparing older versus younger DNAm age groups revealed a statistically significant increased incidence of infection in the older DNAm age group (59%) compared with the younger DNAm age group (32%) (*P* = 0.04) (Table 4). No association was observed between DNAm age and rejection.

**TABLE 4.**
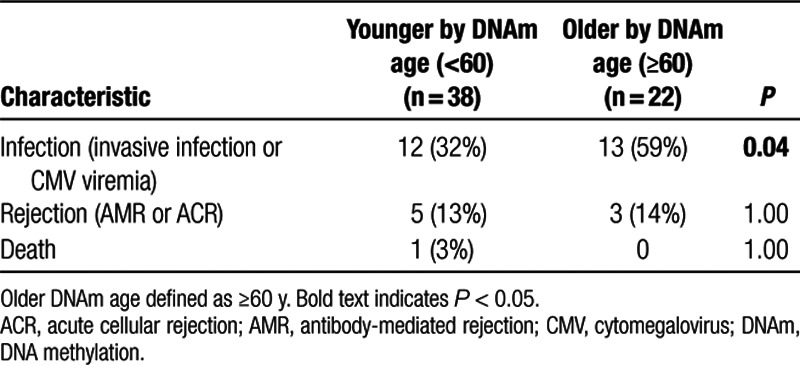
Association between DNAm age and clinical outcomes

**FIGURE 2.**
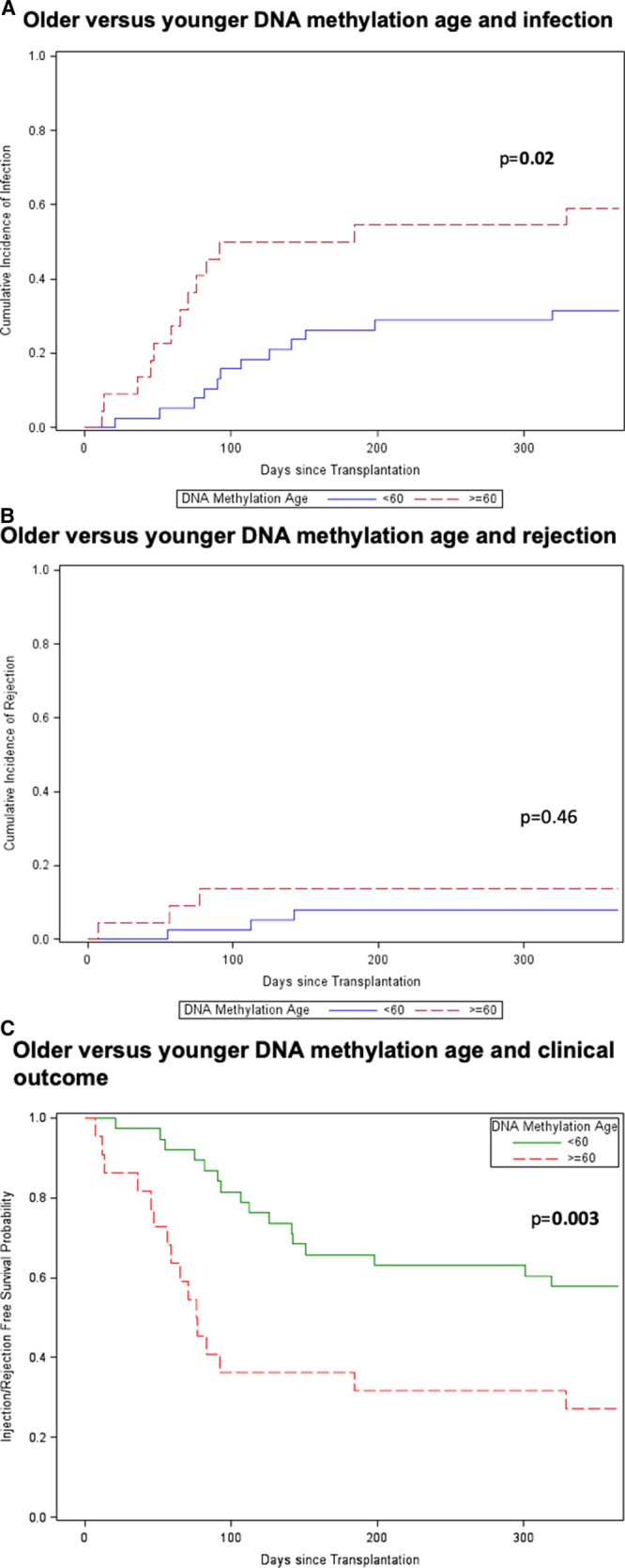
Older compared with younger DNAm age and clinical outcomes. Time to event analysis was performed for older (≥60 y; n = 22) vs younger (<60 y, n = 38) DNAm age groups for infection (*P* = 0.02) (A), rejection (*P* = 0.46) (B), and competing outcomes of infection or rejection (*P* = 0.003) (C). Older patients shown by dotted-red line and younger patients by solid blue or green line. Statistical analysis for competing events was performed using Gray’s test. DNAm, DNA methylation.

Competing risk analysis by DNAm age (< versus ≥60 y) revealed that DNAm age ≥60 years was associated with a significantly greater rate of infection in the first year after kidney transplantation (*P* = 0.02) (Figure 2A). A significant association of rejection with DNAm age was not seen (*P* = 0.46), although this analysis may have been limited by the relatively small number (n = 8) of rejection events in this cohort (Figure 2B). Older DNAm age was also significantly associated with a higher rate of adverse clinical outcomes (infection or rejection) in a Kaplan-Meier survival analysis (*P* = 0.003) (Figure 2C).

**FIGURE 3.**
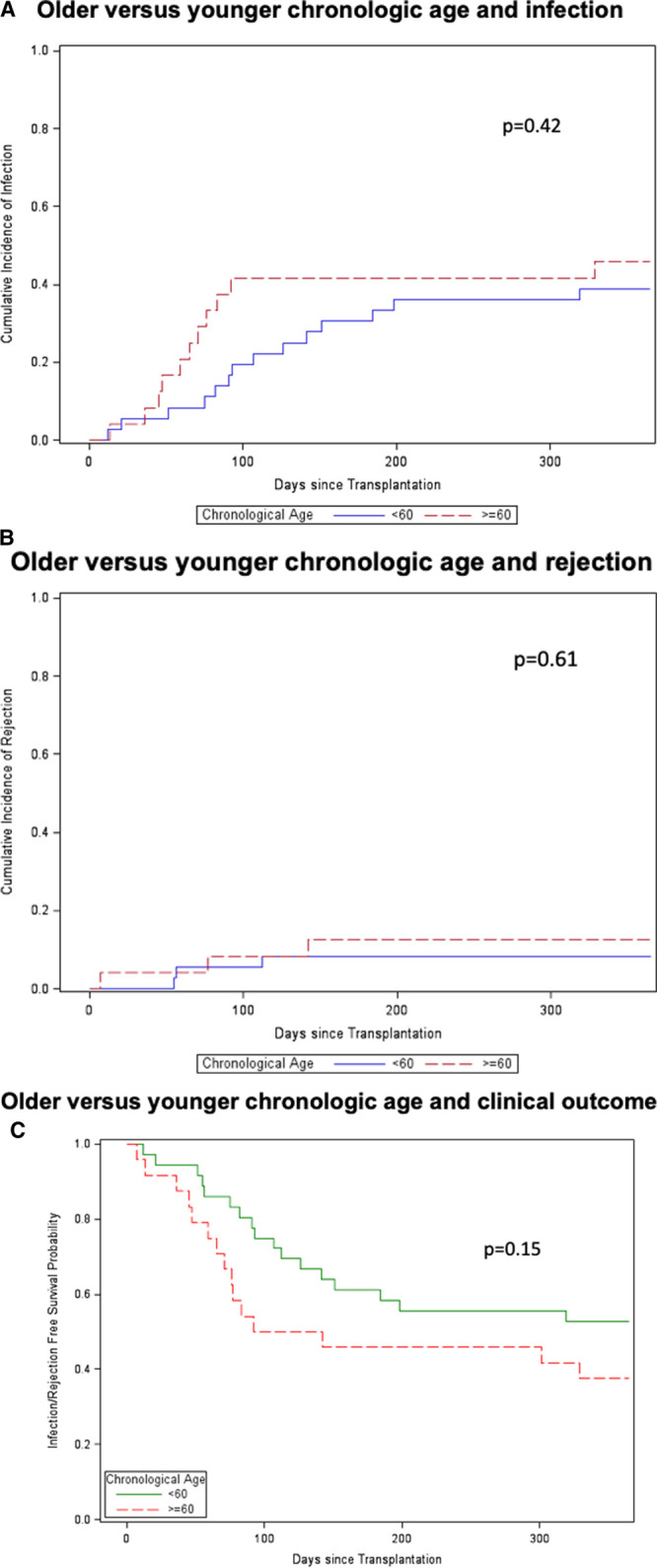
Older compared with younger DNA chronological age and clinical outcomes. Time to event analysis was performed for older (≥60 y; n = 15) vs younger (<60; n = 45) chronological age groups for infection (*P* = 0.42) (A), rejection (*P* = 0.61) (B), and competing outcomes of infection or rejection (*P* = 0.15) (C). Older patients are shown by dotted-red line and younger patients are shown by solid blue or green line. Statistical analysis for competing events was performed using Gray’s test.

Similar time to event analysis for chronological age (< versus ≥60 y) did not demonstrate associations with clinical outcomes of infection (Figure 3A), rejection (Figure 3B), or the combined endpoints (Figure 3C) (*P* = 0.42, 0.61, and 0.15, respectively).

To examine the association between DNAm age (<60 versus ≥60 y) and infection and rejection among patients who have not had an event within 3 months of transplantation, we performed a subgroup analysis by excluding the 28 patients that experienced either infection or rejection within 3 months of transplantation. There was no significant association between either infection (*P* = 0.63) or rejection (*P* = 0.45) and DNAm age (Figure S1, SDC, http://links.lww.com/TXD/A2587); however, this analysis was likely limited by the small patient cohort.

### Calculation and Analysis of DNAm Age Acceleration

DNAm age acceleration residual in the sample ranged from –11.0 years to +17.2 years but was not statistically significant associated with the clinical outcomes of either infection or rejection. Time to event analysis did not reveal an association between positive or negative age acceleration residual and either infection (*P* = 0.82) or rejection (*P* = 0.67) in the first year after kidney transplantation. Results were similar for age acceleration difference (data not shown).

## DISCUSSION

We have applied the concept of the “epigenetic clock” to kidney transplant recipients to test whether DNAm age calculation could be successfully performed on PBMC in posttransplant patients on immunosuppression. In this pilot study, we found an association between DNAm age, but not chronological age, and incidence of infection in kidney transplant recipients, suggesting the potential application of this analysis for risk stratification by calculation of biological age. We were successfully able to perform DNAm analysis and calculate DNAm age on a cohort of 60 older and younger kidney transplant recipients. This analysis revealed an overall linear association between chronological age and DNAm age, however, many patients demonstrated age acceleration, or increased DNAm compared with chronological age (Figure 1). This suggests, as suggested by the differential clinical outcomes experienced by patients undergoing similar medical procedures, that many patients may be at a biologically advanced age immunologically compared with their chronological age. Conversely, chronologically older patients may be biologically younger and expected to demonstrate a clinical trajectory similar to a younger patient.

DNAm age did demonstrate a significant association with overall incidence of infection in the first year after kidney transplantation, both by overall incidence and by time to event analysis. That an association was seen even in this relatively small pilot study suggests the promise of this measure of biological as opposed to chronological age for patient risk stratification and individualization of immunosuppression regimens. The lack of association between DNAm age and rejection may reflect the low incidence in this cohort, future studies are needed to validate the association seen with infection and explore whether decreased DNAm age can predict increased risk for rejection in a larger cohort with correction for potential confounding variables such as induction type. One limitation of this study was the observation that many clinical events occurred before PBMC analysis so that DNAm profile was measured before or after the infection or rejection event. Future studies are needed to further explore the timing of DNAm age measurement, infection, and rejection.

We additionally explored the idea that positive age acceleration would be associated with development of infection, while in contrast, negative age acceleration, or retardation of DNAm age, would be associated with rejection. However, we found that although DNAm age-predicted outcomes after transplantation better than chronological age, age acceleration (the difference between DNAm age and chronological age) did not predict posttransplantation outcomes.

These preliminary data suggest that calculation of epigenetic or DNAm age holds promise as a marker of biological risk in older and younger kidney transplant recipients. Because PBMC contain the immune cells most important for protection from infection and development of rejection, this biological compartment is an ideal target for assessment of DNAm age that would be predictive of relevant posttransplant outcomes. DNAm age may be a valuable addition to other well-characterized clinical variables known to be associated with clinical outcomes such as presence of donor-specific antibodies and recipient comorbidities such as diabetes mellitus.

Application of DNAm age to risk stratification for transplant recipients has been suggested as potentially beneficial by many authors,^21-23^ but to our knowledge, this the first report of this analysis being performed in solid organ transplant recipients. In the nontransplant literature, there are many reports demonstrating the ability of DNAm age acceleration to predict development of age-associated morbidities and mortality.^12,13,17,24,25^ This method has also been applied to the field of cancer biology, where changes in DNAm and age acceleration of premalignant tissue is associated with development of malignancy.^26,27^ These previous reports add to the promise of measurement of DNAm age and age acceleration as a potential tool for candidate assessment and risk stratification after transplantation. Assessment of DNAm age of PBMC may be a better guide to adjustment of immunosuppression such as minimization of ATG or tacrolimus dosing, as has been suggested with varying degrees of success based on patient chronological age.^28,29^ DNAm age may complement the measurement of physical frailty, which has attracted interest as a potential approach for evaluation of transplant candidates.^30^

In addition to having potential as a tool for medication adjustment, analysis of DNAm age acceleration can provide insights into mechanism behind clinical outcomes. Given that the most populous cell within PBMC is the T cell, it is likely that the age acceleration observed reflects T-cell immunosenescence, which is known to be associated with increased vulnerability to infection and relative protection from rejection.^8,31,32^

The limitations of this study include its cross-sectional design and the testing at a single posttransplant time point, as well as the relatively small size of the patient cohort. Patients studies are often limited by heterogeneity of the patient cohort and the single episode of DNAm age testing, but this is somewhat meliorated because of the single-center study design and use of identical protocols for immune suppression across all patient groups. We were also limited by the small number of rejection complications, which may have affected our ability to determine an association between DNAm age and rejections. Furthermore, it is possible that episodes of infection may have impacted DNAm age.

Future directions will include validation of study findings in a larger patient cohort, which will allow closer examination of the possible association between positive DNAm age acceleration and infection, and conversely negative DNAm age acceleration and rejection. In addition, we will evaluate pretransplant DNAm age to determine the impact of transplantation and start of immunosuppression on DNAm age acceleration in older and younger kidney transplant recipients, before any episodes of infection or rejection. We will additionally perform genome-wide association study to determine whether we can identify a subset of CpG sites, which are differentially methylated by patient age, pretransplant comorbidities, and clinical outcomes. In future clinical studies, immunosuppression could be reduced, for example, by reducing mycophenolate mofetil dose in patients with older DNAm age to determine whether this dose reduction can lead to decreased rate of infection compared with patients receiving standard medication doses.

Evaluation of DNAm age by analysis of methylation state of PBMC is a noninvasive method for assessing a patient’s biological or immunological age, a measurement, which for some older patients may be significantly higher or lower than their chronological age. Incorporation of this assessment into evaluation of solid organ transplant candidates and recipients holds promise for improving clinical assessment and individualization of immunosuppression regimens and should shed light into the mechanism of vulnerability of older transplant patients to infection and death.

## Supplementary Material


