# Supplementary material for: DNA Methylation Age Is More Closely Associated With Infection Risk Than Chronological Age in Kidney Transplant Recipients
Source: Transplant Direct. 2020 Jul 15;6(8):e576. doi: 10.1097/TXD.0000000000001020 (PMC7581059; doi:10.1097/TXD.0000000000001020)
Supplement: Supplementary file 1 [file txd-6-e576-s001.pdf]

FIGURE S1. Older compared with younger DNA methylation (DNAm) age and clinical outcomes, restricting outcomes to those occurring 3 months posttransplant. Time to event analysis was performed for older (≥60 years) versus younger (<60 years) DNAm age groups for infection ($p=0.63$) (A), rejection ($p=0.46$) (B), and competing outcomes of infection or rejection ($p=0.94$) (C). Older patients shown by dotted red line and younger patients by solid blue line. Statistical analysis for competing events was performed using Gray's test.

A.

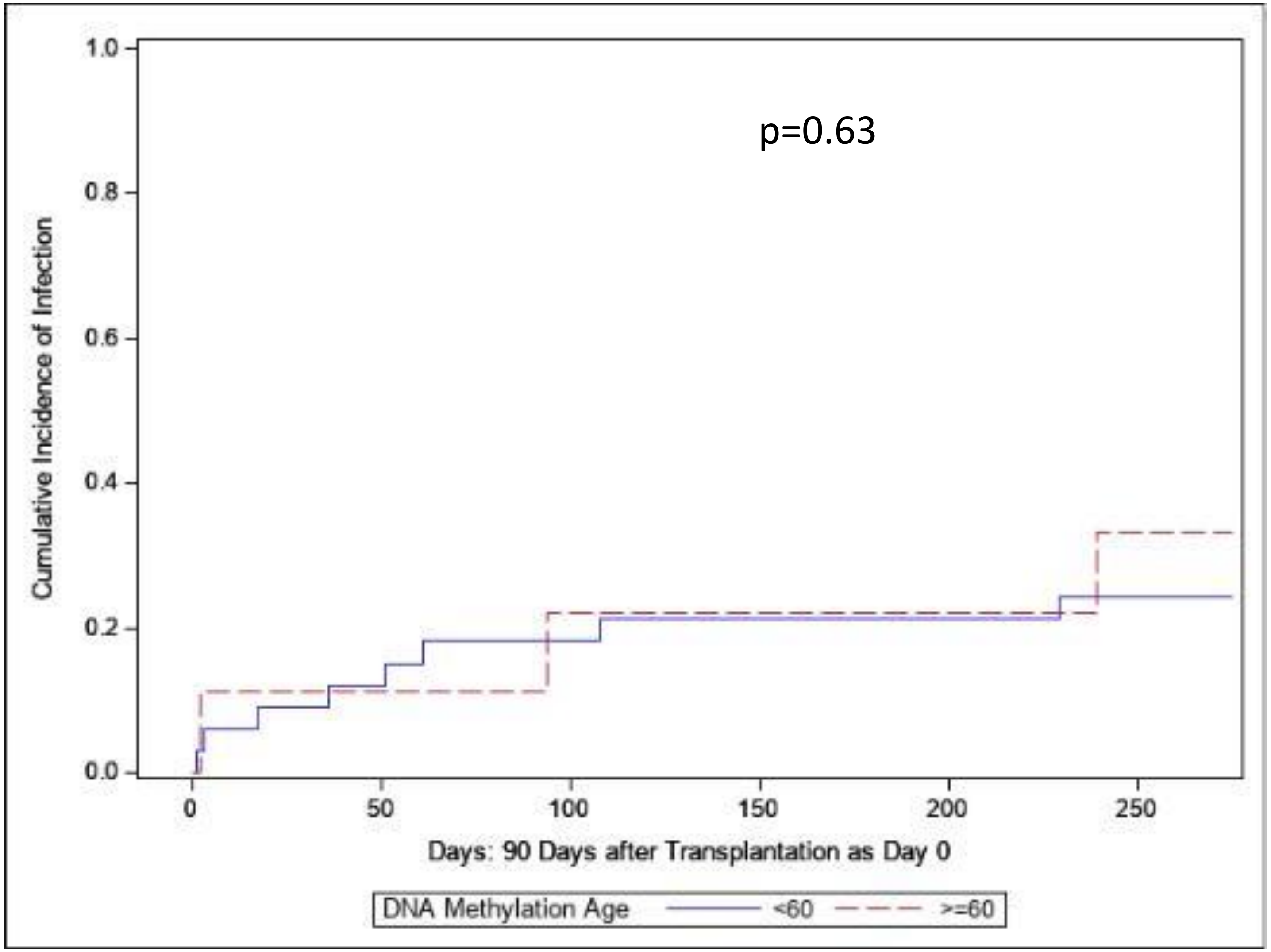

p=0.63
1.0
0.8
0.6
0.4
0.2
0.0
Cumulative Incidence of Infection
0
50
100
150
200
250
Days: 90 Days after Transplantation as Day 0
DNA Methylation Age
<60
>=60

B.

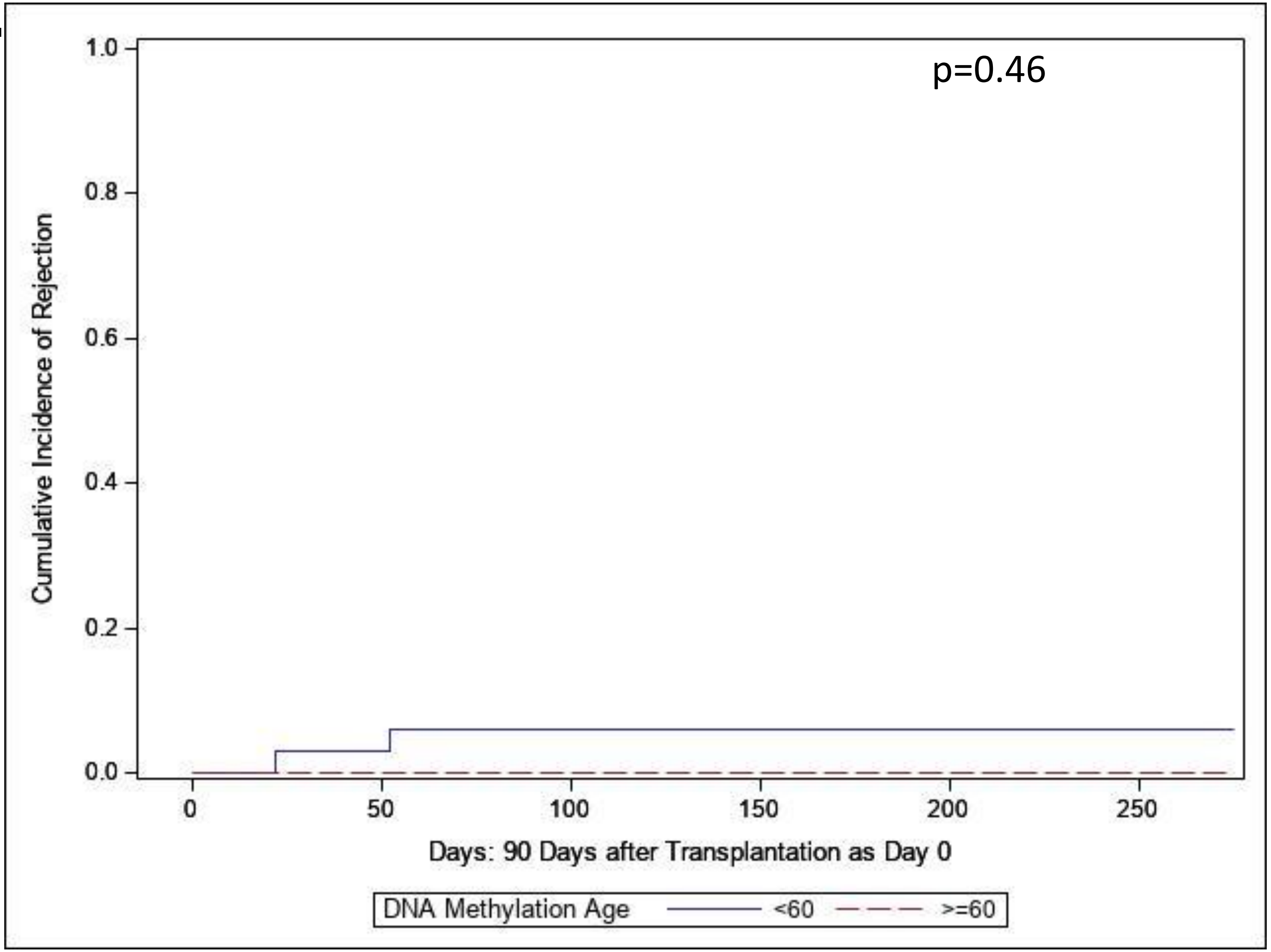
p=0.46
1.0
0.8
0.6
0.4
0.2
0.0
Cumulative Incidence of Rejection
0
50
100
150
200
250
Days: 90 Days after Transplantation as Day 0
DNA Methylation Age
<60
>=60

C.

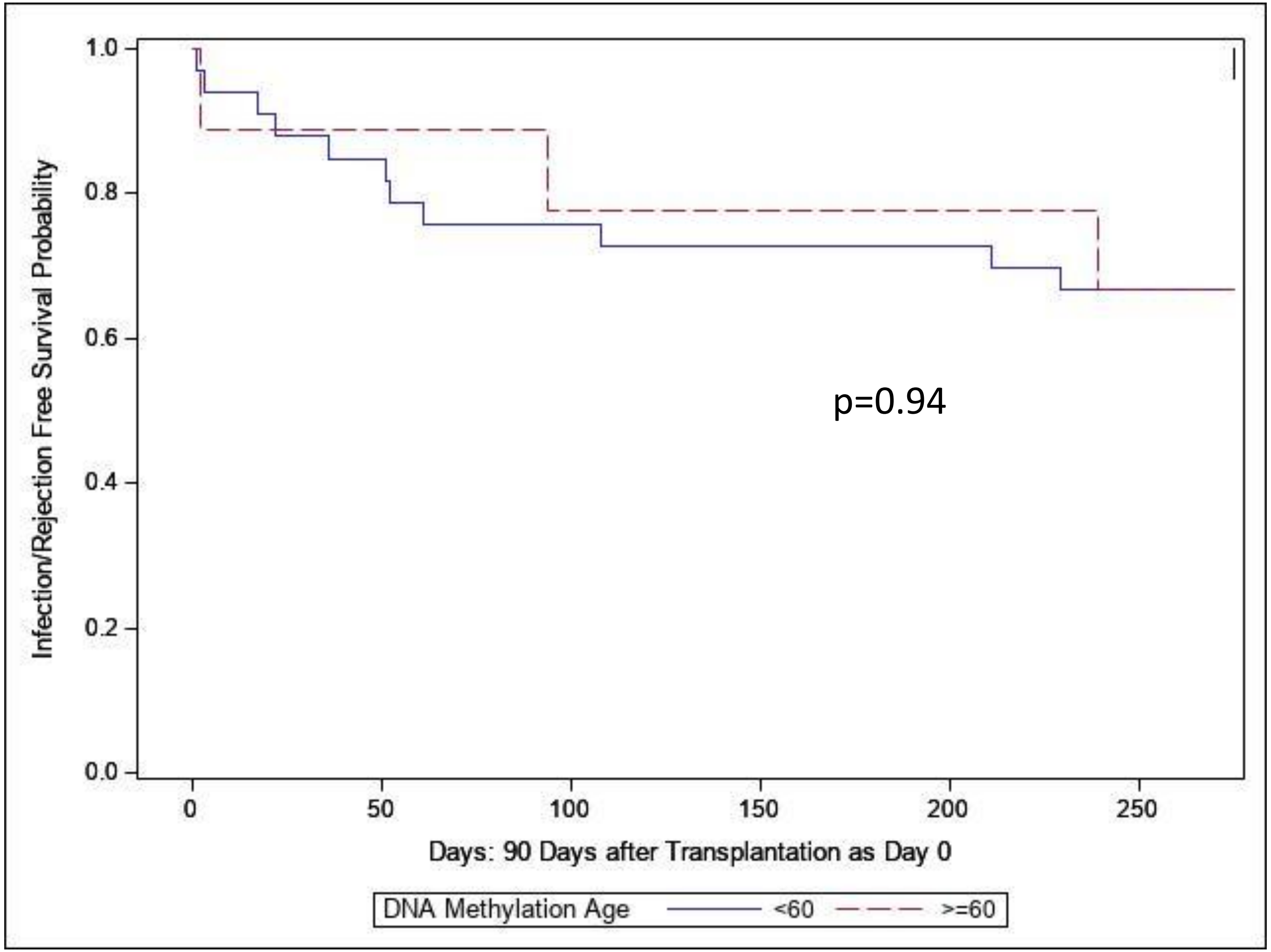
1.0
0.8
0.6
0.4
0.2
0.0
Infection/Rejection Free Survival Probability
0
50
100
150
200
250
Days: 90 Days after Transplantation as Day 0
p=0.94
DNA Methylation Age
<60
>=60
